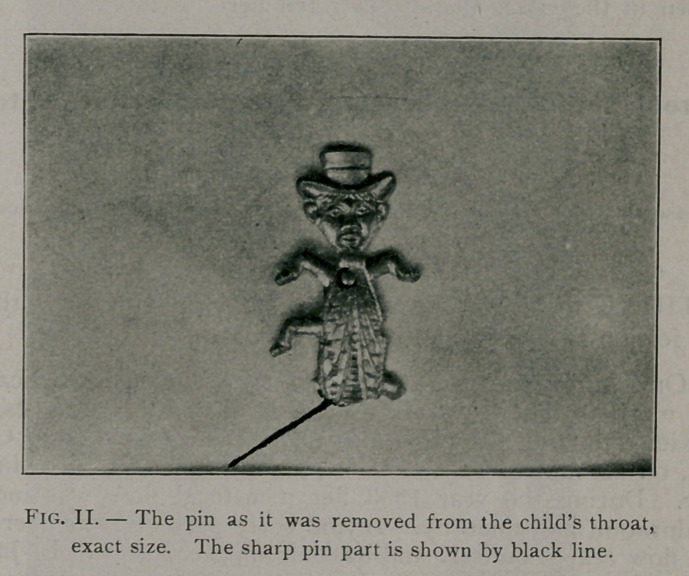# From the Surgical Clinic at the Buffalo General Hospital

**Published:** 1905-02

**Authors:** 


### CLINICAL REPORTS.

### From the Surgical Clinic at the Buffalo General Hospital.

### CONTRIBUTION TO THE LITERATURE OF FOREIGN BODIES IN THE PHARYNX AND ESOPHAGUS.

EARLY in December, 1904, a little girl, 18 months of age, was brought to the surgical clinic of the general hospital by frightened parents, who stated that she had swallowed, or, at least, had passed down her throat beyond reach, a toy stick pin, "shaped somewhat like the picture of a doll," with a long pin in the white metal part which represented the body of the doll itself. She had sustained this accident some two hours before she reached the clinic, at which time she was suffering very slight embarrassment of respiration with inability to swallow, but did not complain very much, except when this effort was made.

In a general way it was learned that the pin was an inch and a half or so in length, of irregular shape and made of flexible white metal. A radiograph, at once made by Dr. Plummer, gave the accompanying picture, showing apparently that the pin was impacted in the throat and esophagus, with the head downward.

Fig. I.—Radiograph, showing location of pin in the child's throat, and its relative size.

The sharp pin part seemed to be shown in the picture projecting upward in such a way as to complicate the effort to disengage in removing it.

The child was thoroughly scrubbed and prepared for external operation, if need be; then chloroformed and placed upon the table with a sand bag under her neck, the head being thrown backward. Under the relaxation of complete anesthesia, the mouth was widely opened with an O'Dwyer mouth gag, and Dr. Park was then able to feel the upper end of the metal object just below the level of the tip of the epiglottis.

He selected forceps with blades bent at right angles to the handle portion, and passing this down behind the tongue and epiglottis, was able, after some effort, to disengage and finally withdraw the object almost entire. In the first efforts that were

FIG. II. — The pin as it was removed from the child's throat, exact size. The sharp pin part is shown by black line.

made, one, or perhaps two, of the projecting portions of the body of the pin were broken loose, and one of them was withdrawn. This will account for the irregularity of the figure shown in the photograph.

Very little blood was lost during the brief operation. The exigency of the case calling for both the surgeon's finger and forceps in the throat, there was caused considerable embarrassment of respiration, and at one time the child became quite cyanosed.

Dr. Park called attention to the fact that one of the immediate dangers in such cases was edema of the glottis, and he directed that ice be applied to the neck externally, and at the same time authorised the house surgeon to do intubation or tracheotomy at any moment, should urgent or distressing symptoms require it. The subsequent course of the case was so uneventful, that nothing of this kind was called for, and the child was taken home on the second day, apparently able to swallow with very little difficulty.

The accompanying figure illustrates the exact size of the object with the pin which, fortunately, had been somewhat loosened, probably before the child swallowed it; this fact having made it easier to disengage than otherwise would have been possible.

It was photographed with the painted side up in order to illustrate the exact nature of the toy which the child was allowed to put into its mouth. One entire limb and part of another have been broken off; just when, cannot be told, but the pin was withdrawn in the exact shape illustrated here.

## Figures and Tables

**Fig. I. f1:**
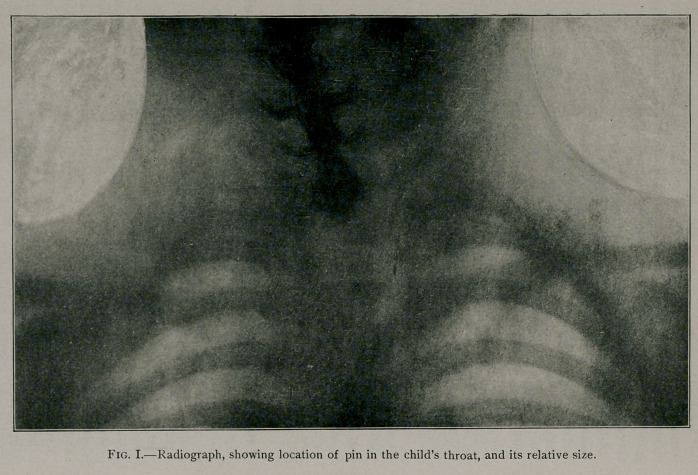


**Fig. II. f2:**